# Interactive Effects of Three Core Goal Pursuit Processes on Brain Control Systems: Goal Maintenance, Performance Monitoring, and Response Inhibition

**DOI:** 10.1371/journal.pone.0040334

**Published:** 2012-06-29

**Authors:** Elliot T. Berkman, Emily B. Falk, Matthew D. Lieberman

**Affiliations:** 1 Department of Psychology, University of Oregon, Eugene, Oregon, United States of America; 2 Institute for Social Research, Departments of Communication Studies and Psychology, University of Michigan, Ann Arbor, Michigan, United States of America; 3 Department of Psychology, University of California Los Angeles, Los Angeles, California, United States of America; University of Bologna, Italy

## Abstract

Goal attainment relies in part on one’s ability to maintain a cognitive representation of the desired goal (*goal maintenance*), monitor the current state vis-à-vis the targeted end state and remain vigilant for lapses in progress (*performance monitoring*), and inhibit counter-goal behaviors (*response inhibition*). Because neurocognitive studies have typically examined these three processes in isolation from one another, little is known regarding if and how they interact during goal pursuit. However, these processes frequently co-occur during online, real-world goal pursuit. The present study employed a novel task to investigate how goal maintenance, performance monitoring, and response inhibition interact with one another. We identified functional activations distinct to each of the processes that correspond to results of prior investigations. In addition, we report interactive effects between response inhibition and goal maintenance in the dorsal anterior cingulate cortex and between performance monitoring and goal maintenance in the superior frontal gyrus and supramarginal gyrus. Implications for studying the neural systems of *in situ* goals include the need for both experimental designs that distinguish between process, but also more complex, realistic tasks to begin to map interactions among these neurocognitive processes and how they are altered by the presence or absence of one another.

## Introduction

The ability to pursue complex, long-term behavioral goals is one of the hallmarks of human behavior. Nearly all adults can recall a time when they’ve set a goal for themselves–to exercise more, to eat less, or to give up a bad habit. To be sure, we often falter in our pursuit. Given the high level of planning and coordination necessary to pursue these goals, it is amazing that anyone is ever successful. The present study seeks to explore the neural systems that allow us to succeed in our goals, and to better understand how these systems may change when deployed in combination with others.

One way of conceptualizing goal pursuit is as a coordinated suite of basic neurocognitive processes. For example, dieting may involve planning meals, resisting impulses to eat unwanted foods, and periodically monitoring ones progress toward a predetermined endpoint. Research has found that individuals who deliberate on the intermediate steps of goal pursuit such as planning and anticipating roadblocks are more likely to succeed in their goals compared to people who focus only on the outcome [1], [2]. Another advantage of unpacking goal pursuit into its basic components is that each one is easier to study alone than the entire process together. For instance, it is far easier to study discrete responses to tempting food in the laboratory than it is to study dieting as a whole. Studying component parts is also easier in a neuroimaging environment such as functional magnetic resonance imaging (fMRI).

For these reasons, much of the research on goal pursuit has focused on basic component processes. We reviewed several models of goal pursuit put forth by social and cognitive psychologists and identified several core components that were common to each [3]. These components include (but are by no means limited to) processes such as goal maintenance, performance monitoring, and response inhibition. *Goal maintenance* refers to maintaining the cognitive representation of a goal or desired end state in working memory at least during a period of goal pursuit (e.g., be friendly during a brief interview for a job), and possibly much longer (e.g., being a nice person over the course of one’s life). *Performance monitoring* refers to being aware of ones current status vis-à-vis the desired end state, evaluating progress, and adapting performance to contextual demands. For example, a dieter may continuously monitor his food intake during meals and also monitor his weight intermittently between meals. *Response inhibition* refers to preventing prepotent or habitual responses that are counter to the goal, or stopping these responses once they’ve begun to occur. Studies using a variety of behavioral and self-report methods have demonstrated that each of these components relates to ultimate goal outcomes ranging from response times and error rates to academic performance and abstinence among drug abusers [4]–[9].

Separately, other research has identified the brain systems recruited during goal pursuit or goal-related processes using neuroimaging tools such as fMRI. This research has identified a set of regions that are consistently implicated in and commonly co-active during top-down control broadly including dorsolateral prefrontal cortex (DLPFC), ventrolateral prefrontal cortex (VLPFC), dorsal anterior cingulate cortex (dACC), and parts of the striatum [3], [10]–[11]. Beyond these broad similarities, goal maintenance, performance monitoring, and response inhibition each recruit a more specific subset of cognitive control regions. We review each below.

### Goal Maintenance

Goal maintenance refers to the ability or capacity to maintain a cognitive representation of the goal in working memory long enough to be able to act on the goal. Though many executive control tasks require goal maintenance, few require it exclusively. For example, goal maintenance is necessary but not sufficient to succeed on the go/no-go task. In the classic version of this task, participants are presented with a string of letters and are instructed to push a button for every letter except “X”, which is presented infrequently. Participants thus have two goals: to push a button for non-X letters, and to withhold a button press for Xs. Two processes are critical to overcome this difficulty: maintaining the dual goals for the task and also, on some trials, inhibiting the pre-potent response to press the button (or, alternatively, top-down biasing the “no-push” goal representation to a greater degree than the “push” representation).

Experimental work on the color-word Stroop task [12]–[13], which also requires goal maintenance and typically activates both the dorsolateral prefrontal and anterior cingulate cortices, has uncovered how these regions separately contribute to task performance. An experiment by MacDonald, Cohen, Stenger, and Carter [14] dissociated the brain regions involved in the two parts of the task (goal maintenance, response inhibition) by inserting a delay between the instructions and the word presentation. In the first phase, participants were instructed to pronounce either the word (“word” trials) or the color (“color” trials) of the upcoming stimulus. After a pause of a few seconds, the stimulus was presented in the second phase. The first part of the task required only the maintenance of the trial-specific goal, and the second part of the task required top-down inhibition during the incongruent color trials. Consistent with other evidence regarding rule-based processing [15]–[16], the dorsolateral PFC was active only during the first portion of the task. Other recent findings have sharpened these conclusions by separating the brain activations related to maintenance of the task goal from those related to processing the visual stimuli of the task. By employing several versions of the Stroop task across multiple stimulus types (e.g., pictures, words presented visually, words presented aurally), Banich and colleagues [17]–[18] found that regions in dorsolateral PFC and inferior parietal cortex are involved in the Stroop task independent of stimulus modality. Another study reported increased activity in lateral parietal regions during maintenance of rules during a visual response contingency task [19]. These studies converge on the finding that the dorsolateral PFC and parietal cortex are involved in maintaining a representation of the goal during the Stroop task, consistent with the role of these frontoparietal regions in working memory processes more generally [20].

### Performance Monitoring

Performance monitoring is an umbrella term that refers to a number of related processes including vigilance to conflict, error detection, and performance adjustment. In the context of goal pursuit, performance monitoring can usefully be sub-divided into one process that detects *goal discrepancy*–the gap between the current state and the desired end state–and another process that dynamically adjusts behavior accordingly to reduce it. Much of the neuroscience research on performance monitoring has focused on disentangling these two processes (i.e., goal discrepancy *detection* from *reduction*). The dACC was initially implicated in both processes [21]–[23], but subsequent work has begun to specify the differential role of the ACC. By manipulating the frequency of incongruent trials in a Stroop task (e.g., “RED” in blue ink), Carter and colleagues [24] found that the dACC was more active when responding to incongruent trials within a context of mostly congruent trials (high detection and low reduction) compared to mostly incongruent trials (low detection and high reduction). This result is in line with a conflict monitoring account whereby the ACC signals a discrepancy and recruits other regions to engage discrepancy reduction processes [22]. Following this, in a Stroop task ACC activation on a previous trial is associated with faster correct responses and, importantly, increased prefrontal activation on subsequent incongruent trials [25], suggesting that discrepancy detection is linked with discrepancy reduction in a dynamic fashion. Hence, researchers use the term “performance monitoring” to refer to instances when both processes might be engaged together to adaptively adjust behavior to meet contextual demands [26]. These processes are likely subserved by an interacting between medial (ACC, anterior insula) and lateral (ventral and dorsal PFC) regions that roughly correspond to detection and reduction, respectively.

### Response Inhibition

There is broad consensus that right inferior frontal gyrus (rIFG) is involved in response inhibition [27]–[29]. Using converging evidence across several inhibitory tasks (e.g., task switching, go/no-go, stop signal), Aron and colleagues have suggested that, although dorsolateral PFC, ventral PFC, and ACC are each activated in tasks that involve inhibition, only the right inferior frontal gyrus (ventrolateral PFC) is *necessary* for inhibition [30]. The causal connection is supported by lesions studies showing that damage to rIFG leads to selective deficits in inhibition [31]–[32]. Further, the personality trait of impulsiveness (or the related construct of novelty seeking) has been linked to reduced activation in ventral aspects of the PFC during inhibition [33]–[34].

More recently, novel techniques have been used to elucidate the network of regions interconnected with rIFG that are also involved in response inhibition. For example, researchers using diffusion tractography identified an inhibition-related fronto-striatal pathway that includes rIFG, the presupplementary motor area (preSMA) and the subthalamic nucleus [35]. The finding that subcortical structures may be involved in inhibitory control is relatively new, but has been supported by other work. In addition to rIFG, recent findings have frequently implicated the anterior insula and parts of the striatum, particularly the head of the caudate, in inhibitory processes across several common tasks [36]–[38]. The involvement of a frontostriatal network in response inhibition makes sense neuroanatomically because of the close interconnections between the ventral striatum and motor regions such as preSMA, SMA, and primary motor cortex [39]. Nonetheless, the precise role and boundary conditions of these subcortical regions in response inhibition remains unclear. For example, there has been some recent debate about whether rIFG is directly involved in inhibiting behavior or another, often confounded, process such as attention to goal-relevant cues [40], top-down response control [41], or expectancy violations [42]. It has recently been proposed that two distinct subdivisions of rIFG, one more dorsal and more ventral, explain the involvement of the rIFG in this variety of processes [43]. One important direction is to explore possible moderating factors (e.g., performance monitoring or goal maintenance demands) of neural activity during response inhibition.

### The Current Study

Despite the progress that has been made in understanding the brain regions involved in goal maintenance, performance monitoring, and response inhibition, there are still a number of unanswered questions. The present study focuses on one important question that is relevant to how people pursue goals *in situ*: How do these processes interact with one another when they co-occur? Because most studies examine only one component in isolation (cf. [41], [44]), whether and how these components interact is largely unknown. Understanding these interactions is critically important because they nearly always co-occur during everyday goal pursuit. People pursue multiple goals simultaneously, and any instance of goal maintenance, performance monitoring, or response inhibition is likely to occur in the context of other goals and cognitive demands. For example, cigarette smokers who seek to quit smoking must simultaneously maintain a cognitive representation of their cessation goal, monitor and adjust their behavior to meet dynamic situational demands (e.g., being tempted to smoke during a work break), and, on occasion, engage inhibitory control against cravings and habitual smoking behavior. One goal of the present study was to directly test whether and how the neural regions implicated in each goal process are altered by the presence of others. In other words, we sought to test the “pure insertion” assumption–that inserting a new mental process to a task does not alter the other, ongoing processes–in the context of goal pursuit [45].

To do this, we created a novel version of the go/no-go task that allows goal maintenance, performance monitoring, and response inhibition to be independently manipulated in order to examine the interactions among them. In the classic version of this task, participants form a pre-potent response to quickly press a button in response to each of a series of letters displayed on a screen (“go” trials). Less than 20% of the time, the participant must withhold a button press to a target letter (“no-go” trials). Brain activation on no-go trials compared to go trials is thought to reflect a combination of response inhibition and conflict detection [46]. We also included blocks of trials where the instruction to “go” by either pulling or pushing a lever (which alternated across blocks) was or was not displayed on the screen. We reasoned that goal maintenance demands were greater on blocks when the variable instruction (“push” or “pull”) was not displayed on the screen compared to blocks when the instruction was always displayed. Finally, in addition to the standard go/no-go blocks, we also included blocks of only go trials (excluding no-go trials). Importantly, participants were informed at the beginning of each block whether or not that block included no-go trials. We reasoned that performance monitoring demands were greater for go trials on blocks that included no-go trials compared to go trials on blocks without no-go trials, because no performance monitoring was required during the all-go blocks.

## Methods

### Participants

Thirty-one right-handed participants (15 female) were recruited from the Los Angeles community via flyers and Internet advertisements to participate in an fMRI study. Their ages varied from 28 to 69* (*M* = 46.0, *SD* = 9.7), and they were ethnically diverse: 52% were Caucasian, 26% Hispanic, 19% African American, and 3% other/declined to report. [^*^ *Note: The effect of age on neural activity was controlled by entering age as a covariate of no interest in all models. The results changed slightly, though not substantively, when age was not controlled.*] Participants were excluded if they were left-handed, did not speak English, consumed more than 10 alcoholic drinks per week, or had any of the following conditions: dependence on substances currently or within one year of the scan date, neurological or psychiatric disorders, cardiovascular disease, pregnancy, claustrophobia, or any other condition contraindicated for MRI (e.g., metallic implants). Participants were compensated $80 at the end of the session. All participants provided written informed consent approved by the University of California, Los Angeles Institutional Review Board (IRB).

### Procedure and Materials

Participants were screened for exclusion criteria via a phone interview one week prior to the scan. Upon entering the lab, participants gave informed consent, were instructed in the task, and performed a computerized training in the task. Participants were also instructed in other tasks that will not be discussed here. Next, participants were situated in the scanner for the duration of the scan. Foam padding was placed around participants’ heads to reduce motion. Stimuli were presented on LCD goggles, and responses were recorded on a magnet-safe joystick placed in the right hand (Resonance Technology, Northridge, CA, USA). Following completion of the task participants were removed from the scanner, debriefed, and compensated.

**Figure 1:**
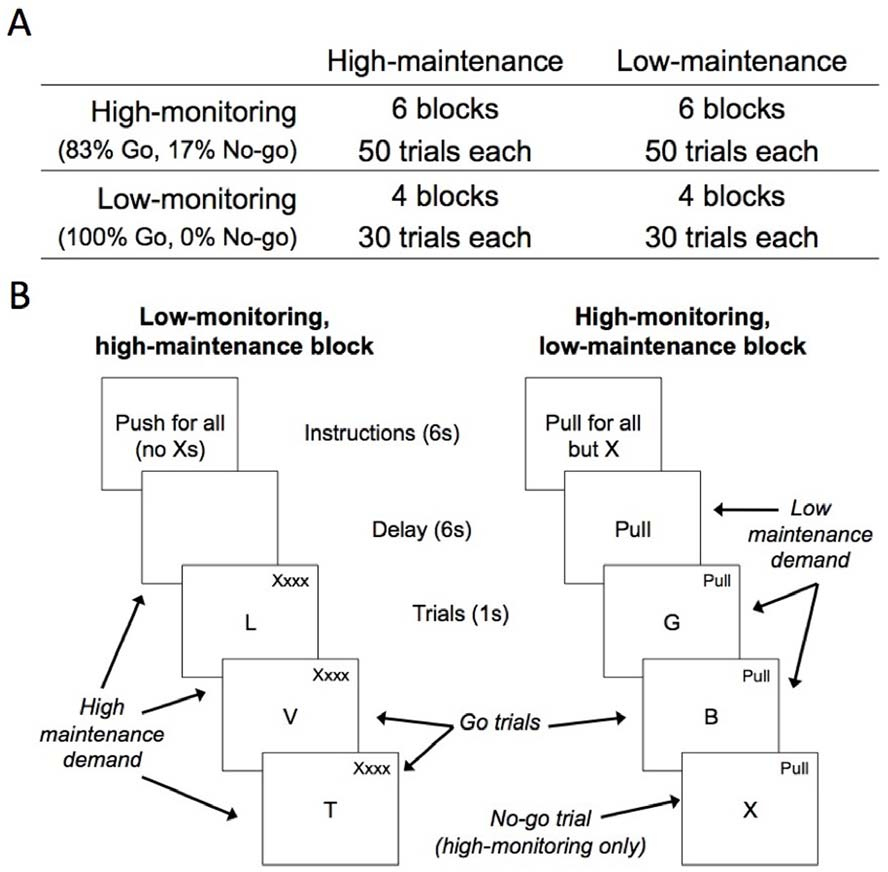
Design of the modified go/no-go task. (A) Block and trial frequencies of the Monitoring (2: high/low) × Maintenance (2: high/low) × Response Inhibition (2: go/no-go) with response inhibition nested within high monitoring. (B) Example blocks for low-monitoring, high-maintenance (left) and high-monitoring, low-maintenance (right). Monitoring was manipulated by the inclusion (high) or exclusion (low) of no-go trials within a block. Maintenance was manipulated by the presence (low) or absence (high) of instructions throughout the block.

We used a modified go/no-go task to examine the neural activation associated with goal maintenance, performance monitoring, and response inhibition (Figure 1). A classic go/no-go task was used on some blocks to assess response inhibition. These blocks contained a series of brief trials each depicting a single letter centered in the screen. Each block began with the instruction to “push” or to “pull” the lever. Participants responded to the trials by pushing or pulling the lever according to the instruction displayed before each block (“go” trials) to the letters L, N, T, and V (∼82% of trials) and withholding a response (“no-go” trials) to the letter X (∼18% of trials). Response inhibition was considered to be engaged on the no-go trials compared to the go trials. Some blocks contained only go trials (i.e., 100% go, 0% no-go), and participants were informed of this at the beginning of the block. Because participants were not required to be vigilant for no-go trials, we considered performance monitoring demands to be reduced on these blocks (“low-monitoring”) compared to blocks that contain both no-go and go trials (“high-monitoring”). Finally, on some blocks the instruction to “push” or to “pull” for each go trial was displayed in the top-right corner of the screen on all trials throughout the block (“low-maintenance”); on other blocks the instruction was not displayed on the screen except during the instruction period at the beginning of the block (“high-maintenance”). We reasoned that goal maintenance demands were increased when the instructions were not displayed throughout the block compared to blocks in which they were.

The task conditions are summarized in Table 1. The task contained high-monitoring blocks and low-monitoring blocks, and the high-monitoring blocks were over-sampled because they contained the no-go response inhibition trials and the low-monitoring blocks do not. Half of all blocks were high-maintenance (and the other half were low- maintenance). Each high-monitoring block contained an average of 9 no-go trials and 41 go trials; the low-monitoring blocks contained 30 go trials. [*Note: This imbalanced design was selected to maximize the power to detect effects of performance monitoring. To test for statistical trial frequency effects, we re-computed each of the results described below using a completely balanced design by discarding a random subset of the high-monitoring blocks (so there were 8 high- and 8 low-monitoring blocks) and high-monitoring go trials (so there were 30 go trials within both high- and low-monitoring conditions). The results were substantially unaffected–all clusters reported in* *Tables 2* *and* *3* *remain above threshold, and no new clusters emerged.*] Each trial lasted 1 second. Trials in the high-monitoring blocks were jittered according to a random gamma distribution (*M* = 1.5 seconds), and trials in the low-monitoring blocks contained only one trial type and thus jittering was not statistically necessary. Thus, the high-monitoring blocks lasted 75 seconds and the low-monitoring blocks lasted 30 seconds. Blocks were separated by a six-second instruction period followed by a six-second delay in which the instruction either continued to be displayed (low-maintenance) or disappeared (high-maintenance). The twenty total blocks were divided across four functional runs containing five blocks each.

**Table 1:** Behavioral responses to the modified go/no-go task: Means (standard deviation).

Condition	Response time in ms	Distance in pixels	Velocity in pixels per ms	Error rate (no-go trials only)
High-monitoring	547.9^a^ (160.5)	539.1 (37.6)	1.23^c^ (0.34)	4.8 (5.7)
High-maintenance	548.4^a^ (163.5)	540.7 (45.5)	1.25^c^ (0.41)	4.6 (6.0)
Low-maintenance	547.3^a^ (157.5)	537.5 (29.8)	1.21^c^ (0.26)	4.9 (5.4)
Low-monitoring	408.6^b^ (131.8)	527.6 (42.1)	2.96^d^ (1.58)	–
High-maintenance	406.7^b^ (133.0)	530.7 (50.9)	2.96^d^ (1.77)	–
Low-maintenance	410.5^b^ (130.6)	524.6 (35.3)	2.96^d^ (1.59)	–

*Note*. *N* = 31. Different superscripts within a column indicate a significant difference at *p*<.01.

**Table 2:** Main effects analyses for the modified go/no-go task.

Effect	Comparison	Region	x	y	z	Cluster size	t-val
Maintenance	High > Low	Premotor cortex/SMA (BA 6)	−27	−22	73	46	5.02
		Occipital lobe	18	−97	−2	321	7.78
			−15	−94	−2	365	6.79
			−42	−70	−11	70	4.34
	Low > High	*None*					
Monitoring	High > Low	dACC	−12	41	19	54	5.07
		Caudate	15	20	10	34	4.55
			−18	11	19	61	6.14
		Putamen	−27	8	1	29	3.98
		Subthalamic nucleus	−3	−19	−5	89	5.20
		Temporal pole	48	11	−14	43	4.47
		Superior temporal gyrus	−51	−10	4	45	5.21
		Anterior insula	30	11	−14	55	4.59
		Amygdala	36	−1	−20	26	4.36
		Precentral gyrus	−48	−4	49	30	4.23
	Low > High	Supramarginal gyrus	−48	−46	46	29	4.32
			45	−46	49	76	5.15
		Occipital lobe	−6	−70	64	44	4.43
			−24	−70	49	183	4.49
			−15	−97	7	220	7.59
			12	−94	1	127	5.89
Response	No-go > Go	Inferior frontal gyrus	51	14	13	147	7.09
inhibition			42	17	1	421	8.15
		Anterior insula	−36	20	−8	494	9.50
			33	17	4	628	8.76
		dACC/preSMA	−9	23	31	79	5.09
			0	38	25	128	4.88
			3	11	52	140	6.18
		DLPFC	30	44	25	264	5.37
			42	8	37	240	6.70
			−45	41	25	79	4.99
		Caudate	12	8	10	45	4.58
		Superior temporal gyrus	48	−25	−2	138	5.18
		Supramarginal gyrus	63	−43	13	223	6.46
			−54	−46	34	280	5.61
		Angular gyrus	39	−52	49	322	6.17
			−54	−49	25	101	4.87
		Occipital lobe	−24	−88	4	562	7.46
			39	−88	1	371	8.80
	Go > No-go	Primary motor cortex	−45	−28	55	651	8.92
		Cerebellum	24	−46	−26	44	5.65
		Posterior cingulate	−3	−58	22	44	3.96
		SMA	−12	−22	55	34	4.94

*Note*. *N* = 31. All regions FDR corrected at *p*<.05. dACC  =  Dorsal anterior cingulate cortex; DLPFC  =  Dorsolateral prefrontal cortex; preSMA  =  Presupplementary motor area; SMA  =  Supplementary motor area.

**Table 3:** Interactions for the modified go/no-go task.

Effect	Comparison	Region	x	y	z	Cluster size	t-val
No-go > go	High maintenance > low maintenance	*None*					
	Low maintenance > high maintenance	preSMA	−6	41	40	18	4.10
		Cerebellum	−6	−43	−23	59	4.43
High-monitoring > low-monitoring	High maintenance > low maintenance	Premotor cortex/SMA (BA 6)	−27	−22	73	16	3.51
			15	−10	76	26	3.79
		Supramarginal gyrus	51	−46	55	325	5.05
			−45	−43	58	61	4.24
		Fusiform gyrus	36	−43	−17	150	5.13
			−39	−40	−17	23	4.44
		Middle temporal gyrus	51	−49	−8	193	4.54
		Occipital cortex	−39	−85	−5	595	8.80
			36	−85	−5	819	7.70
	Low maintenance > high maintenance	*None*					

*Note*. *N* = 31. All regions FDR corrected at *p*<.05. dACC  =  Dorsal anterior cingulate cortex; preSMA =  Presupplementary mortor area; SMA  =  Supplementary motor area.

Participants responded to each go trial by pushing or pulling a lever then clicking a button at the top of the lever. Response time was computed as the latency between stimulus onset and the button click, errors were determined according to trial type, and distance and velocity were calculated based on the position of the lever at the time of the button click.

### fMRI Data Acquisition and Preprocessing

Brain imaging data were acquired on a 3 T Siemens Trio scanner at the UCLA Ahmanson-Lovelace Brainmapping Center. High-resolution structural T2-weighted echo-planar images (spin-echo; TR  = 5000 ms; TE  = 34 ms; matrix size 128×128; 34 sagittal slices; FOV  = 192 mm; 4 mm thick) were acquired coplanar with the functional scans. Four functional scans lasting 6∶30, 5∶46, 5∶46 and 5∶00 were acquired during the task (echo-planar T2*-weighted gradient-echo, TR  = 2000 ms, TE  = 30 ms, flip angle  = 90°, matrix size 64×64, 34 axial slices, FOV  = 192 mm; 4 mm thick), totaling 692 functional volumes.

The imaging data were analyzed using a combination of FSL tools (FMRIB Software Library; Oxford University, Oxford, UK) and SPM8 (Wellcome Department of Cognitive Neurology, Institute for Neurology, London, UK). The preprocessing stream for the images was as follows. All images were brain-extracted using BET (FSL’s Brain Extraction Tool) and realigned within runs using MCFLIRT (FSL’s Motion Correction using FMRIB’s Linear Image Registration Tool), then checked for residual motion and noise spikes using a custom automated diagnostic tool (thresholded at 2 mm motion or 2% global signal change from one image to the next). In SPM8, all functional and anatomical images were reoriented to set the origin to the anterior commissure and the horizontal (*y*) axis parallel to the AC-PC line. Also in SPM8, functional images were corrected for slice acquisition timing differences within volumes, realigned within and between runs to correct for residual head motion, and coregistered to the matched-bandwidth structural scan using a 6-parameter rigid body transformation. The coregistered structural scan was then normalized into the Montreal Neurological Institute (MNI) standard stereotactic space and the resulting parameters were applied to all functional images. Finally, the normalized functional images were smoothed using an 8 mm full width at half maximum Gaussian kernel.

One run from each of two participants was removed due to motion. Data from three other participants contained motion spikes that were statistically removed using regressors corresponding to the affected scans.

### Statistical Model for the fMRI Data

The design was modeled within subjects using an event-related 2×2×2 factorial design with the following factors: Maintenance (high/low), Monitoring (high/low), and Response Inhibition (go/no-go) nested within high-monitoring blocks. By definition the no-go (response inhibition) trials occur only during the high-monitoring blocks, and do not occur in the low-monitoring blocks. In other words, it was not possible to cross the Monitoring and Response Inhibition factors. All other factors were crossed resulting in a total of six conditions: high-maintenance high-monitoring go, high-maintenance high-monitoring no-go, high-maintenance low-monitoring go, low-maintenance high-monitoring go, low-maintenance high-monitoring no-go, and low-maintenance low-monitoring go. The 12-second fixation periods that followed each block comprised the implicit baseline, and were not included in any analyses. Each trial was modeled as an event with 1-second duration and convolved with the canonical hemodynamic response. The model used a first-order auto-regressive error structure to account for temporal autocorrelations in the functional data.

The main effects and interactions of the factors were defined using a set of orthogonal linear contrasts among the regressors. Each contrast directly compared two or more of the conditions to one another to test main effects (e.g., all high-maintenance trials vs. all low-maintenance trials for the main effect of maintenance) and interactions (e.g., [high-monitoring no-go > high-monitoring go] > [low-monitoring no-go > low-monitoring go] for the interaction between inhibition and monitoring). The resulting contrast images were averaged across runs for each participant, and then entered into a random effects analysis at the group level for greater generalizability. We used a Monte Carlo simulation (AlphaSim; FSL, Oxford University, Oxford, UK) to determine that the minimum cluster size necessary to maintain a false detection rate of 5% was a voxel-wise threshold of 001 combined with a 16 3×3×3 mm voxel cluster threshold. All functional imaging results are reported in MNI coordinates.

Data based on this sample has been reported elsewhere examining the relationship between response inhibition and real-world outcomes [47], but none of the analyses in the present study have been reported elsewhere.

## Results

### Behavioral Data

The mean response times, velocities, and distances for go trials as well as error rates for each condition are displayed in Table 1. Consistent with the increased demand of the high-monitoring blocks, participants were faster to respond to go trials during low-monitoring than high-monitoring (*M*s  = 409 ms and 548 ms, *SD*s  = 132 ms and 161 ms, respectively, *F*(1, 30)  = 106.109, *p*<.01). In addition to response latency, we also calculated the distance participants pushed the lever on each trial. On average, participants pushed the lever 533.4 pixels from the middle of the screen, and there were no differences among the conditions (all *p*s *ns*). By combining latency and distance, we could calculate movement velocity in terms of pixels per ms. Due to the difference in response latency between low- and high-monitoring, lever movement velocity was also higher during low-monitoring than high-monitoring go trials (*M*s  = 1.29 pixels/ms and 0.98 pixels/ms, *SD*s  = 0.99 and 0.34, respectively, *F*(1, 30)  = 53.19, *p*<.01). There was no difference in response time or velocity between low- and high-maintenance (all *p*s *ns*), suggesting that participants were successfully able to maintain the task rule in working memory without impacting performance. The overall error rate (false positives) on no-go trials was 4.6%. The rates for low- and high-maintenance were 4.3% and 4.9%, respectively (difference *ns*). The rate of omission errors on go trials was at or near 0% for all participants.

Because of the differences in reaction time between high- and low-monitoring trials, all event-related analyses below include response time as a covariate at the first level. The purpose of this is to examine the main and interactive effects of these psychological processes on brain activity while holding performance constant.

### Neuroimaging Data

#### Main effects

The main effects of the three goal pursuit components under investigation are shown in Table 2. To examine the neural correlates of increased goal maintenance demand, we contrasted high- with low-maintenance trials. This comparison revealed activations in premotor cortex/supplementary motor area and occipital cortex, perhaps reflecting increased activation of the motor-related goals of pushing and pulling. The activation in these regions did not differ between no-go and go trials (see below for interaction analyses). No region showed significant increases in low- greater than high-maintenance demand trials.

Next, we examined activation during go trials when they were intermingled with no-go trials (high monitoring) compared to when they were not (low monitoring). As expected, performance monitoring recruited dorsal anterior cingulate cortex (dACC), as well as the right temporal pole, left superior temporal gyrus and precentral gyrus, and several subcortical structures including caudate, putamen, subthalamic nucleus, anterior insula, and amygdala. Activation in this network is consistent with the state of vigilant awareness (dACC, STN, temporal pole, insula, amygdala) and preparation for changes in ongoing motor activity (caudate, putamen, precentral gyrus; see Table 2).

**Figure 2:**
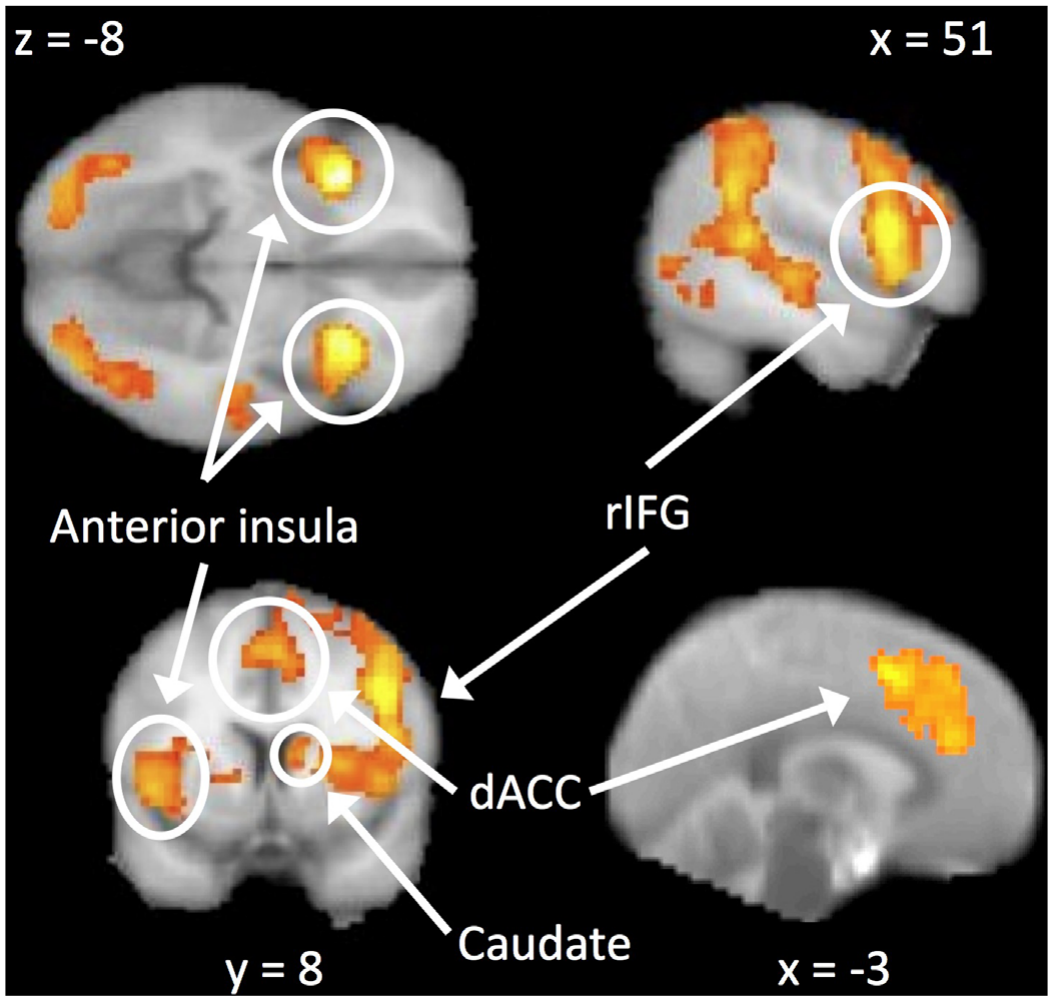
The main effect of response inhibition. Activations were observed in bilateral anterior insula (left images, peak MNI: −36 20 −8 and 33 17 4), dACC (bottom images, −9 23 31), rIFG (top right and bottom left, 51 14 13), and caudate (bottom left, 12 8 10).

Finally, we examined the comparison of no-go to go trials to assess the neural activity associated with successful response inhibition. Also as expected, response inhibition recruited right inferior frontal gyrus, bilateral anterior insula, caudate, and dACC extending into SMA/preSMA (Table 2; Figure 2). We also observed activation in bilateral dorsolateral PFC, inferior parietal lobe (supramarginal and angular gyrus) and occipital cortex. The only activations that were greater during go than no-go trials were left motor cortex and right cerebellum (contralateral and ipsilateral to the right hand response, respectively), supplementary motor area, and posterior cingulate.

#### Interactions

**Figure 3:**
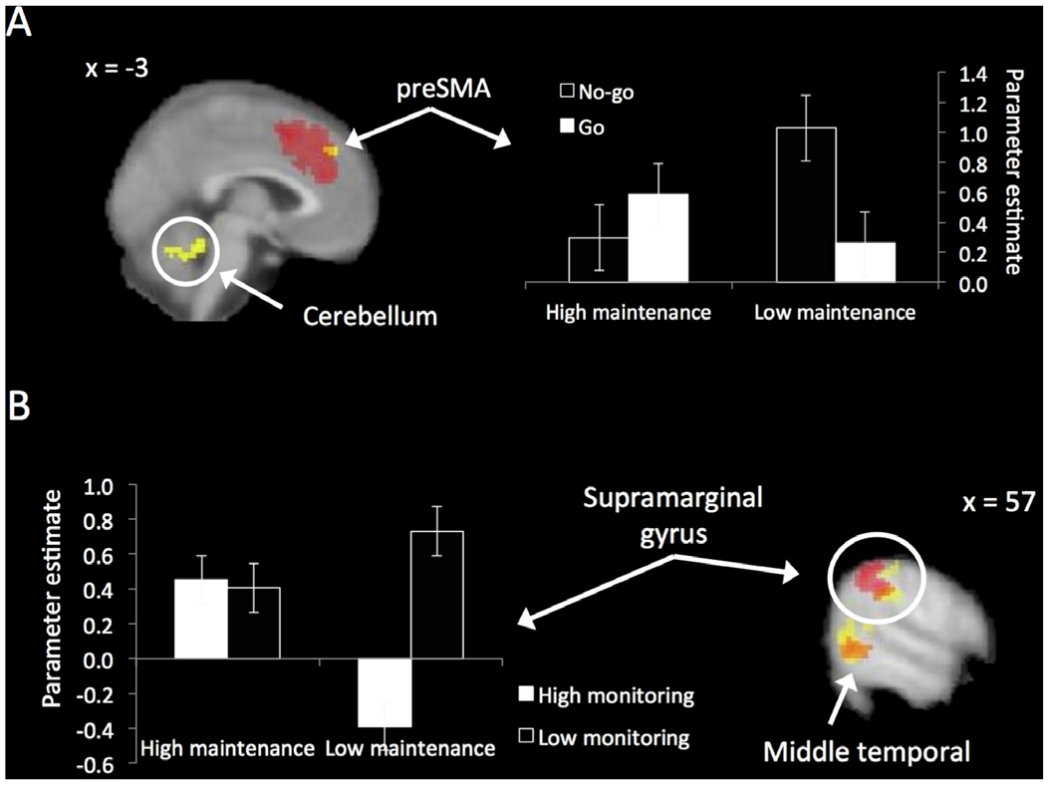
The interactions among the three goal pursuit components. Bar charts represent the average parameter estimate across the entire (yellow) interaction cluster identified in the whole-brain analysis. (A) The interaction between goal maintenance and response inhibition. The preSMA and cerebellum showed increased activation during no-go > go low-maintenance compared to no-go > go high-maintenance (right). The bar chart displays the main effect of response inhibition as well as the two-way interaction in the preSMA. The preSMA interaction activation is shown in yellow overlayed on the dACC/preSMA activation from the main effect contrast of no-go > go in red (left). (B) The interaction between goal maintenance and performance monitoring. The supramarginal gyrus showed increased activation during low-monitoring > high-monitoring for low-maintenance blocks compared to the same contrast for high-maintenance blocks (left). The bar chart displays the main effect of low-monitoring > high-monitoring and the two-way interaction. The supramarginal gyrus activation (yellow) is shown overlayed on the activation from the main effect contrast of low-monitoring > high-monitoring (red).

This design affords the opportunity to examine the interaction among goal pursuit components. The interaction between response inhibition and maintenance tests whether the [no-go > go] response inhibition contrast differed between low- and high-maintenance blocks. In other words, this contrast identifies regions that show a larger inhibition effect during low- than high-maintenance blocks. This interaction was observed in the preSMA and cerebellum (Table 3; Figure 3). Interrogation of the simple main effects (i.e., no-go > go low-maintenance and no-go > go high-maintenance) revealed that activation in these regions was greater during no-go > go low-maintenance than in no-go > go high-maintenance. This was further verified by masking the interaction contrast inclusively with only those regions that showed activation in the simple main effect of no-go > go in low-maintenance blocks. The interacting cluster from the preSMA overlapped almost entirely with the (larger) dACC/preSMA cluster from the main effect of response inhibition (Figure 3). No regions were more active in the opposite contrast. In other words, the effect of response inhibition was particularly high when maintenance demands were low in preSMA (a sub-cluster of that found during response inhibition across all levels of maintenance).

We were also able to examine the interaction between goal maintenance and performance monitoring. Specifically, we compared whether the contrast [high-monitoring > low-monitoring] among go trials was greater during high- compared to low-maintenance blocks. This interaction was observed in premotor cortex and bilateral occipital cortex (Table 3). Examination of the simple main effects revealed that the interaction in these regions was driven by increased activation in the [high-maintenance > low-maintenance] contrast for high-monitoring relative to the same contrast for low-monitoring. In other words, activity in these regions peaked when maintenance and monitoring demands were both high.

We also observed an interaction between maintenance and monitoring in a cluster of regions that showed increased activation in low- relative to high-monitoring demands. These included the supramarginal gyrus and the lateral occipital cortex extending laterally into the middle temporal gyrus and caudally into the fusiform gyrus (Table 3; Figure 3). Inspection of the simple main effects suggested that this interaction was driven by significantly greater activation in these regions during low- relative to high-monitoring in the low-maintenance trials, but no difference in the high-maintenance trials (Figure 3). In other words, these regions are differentially active in low- versus high-monitoring, but only when goal maintenance demands are low.

As noted above, we were unable to compute the interaction between performance monitoring and response inhibition because response inhibition was nested within high-monitoring blocks.

## Discussion

The present study examined the neural correlates of three core components of goal pursuit: goal maintenance, performance monitoring, and response inhibition. The task design allowed for each process to be inspected individually and as it interacted with other processes. This approach enabled us to address unanswered questions about the neural systems that enable complex goal pursuit including how they modulate during co-occurrence with other cognitive processes. The results highlight the importance of studying multiple simultaneous processes, and the dynamic nature of higher-order cognitive phenomena such as goal pursuit.

We manipulated the three components using a modified version of a go/no-go task. Response inhibition was examined in the contrast of no-go trials with go trials. The results from this analysis added convergent validity to the growing consensus that the no-go inhibition network includes rIFG, dACC, anterior insula, dorsolateral PFC, and caudate.

In addition to the standard comparison of no-go to go trials used to assess response inhibition, the task also included factors for performance monitoring and goal maintenance. Performance monitoring was manipulated though the presence or absence of no-go trials within a block. We examined only differences between the go trials within these blocks, and statistically removed activity related to no-go trials. In this comparison, we observed that performance monitoring was associated with increases in dACC, striatum, insula, amygdala, and subthalamic nucleus, among other regions. Several of the regions that were found to be positively associated with performance monitoring–notably the insula, dACC, and STN–are commonly implicated in response inhibition [38]. In fact, we found increased activation in insula, dACC, and caudate during response inhibition in the present experiment. However, in this experiment as in others, the simple no-go > go contrast assessing response inhibition is confounded with performance monitoring because no-go trials require both the detection of a need for control and the engagement of that control [26]. The fact that increased activation in these regions was observed during go trials that were intermingled with no-go trials compared to go trials that were not suggests that the role of these regions in the present task may indeed be more accurately characterized as ongoing monitoring for instances when inhibition may be necessary (i.e., performance monitoring) rather than as inhibitory control per se. This result is in line with recent work showing that activation in preSMA is directly involved in stopping, but that other parts of the inhibitory control network (e.g., anterior insula/rIFG) may be more involved in detecting task-relevant cues to engage in behavioral control or alteration [40]–[43]. This possibility is further supported by the finding that activation in dACC and insula is observed during high- compared to low-monitoring go even when no-go trials (and presumably the engagement of response inhibition) are statistically controlled.

In contrast, we observed that increased performance monitoring was associated with relative reductions in supramarginal gyrus activation. It is interesting to consider this finding in light of a growing body of work implicating the supramarginal gyrus as part of the “default mode” network [48]–[49]. One hypothesis is that supramarginal gyrus activations in the default mode network reflect broad attention during rest, and that attentional “filtering” during a task causes reductions in supramarginal gyrus activation. In support of this notion, one study found that the extent of deactivation in supramarginal gyrus was correlated with performance in a visual search task [50]. The data presented here are consistent with the hypothesis that the supramarginal gyrus activation reflects attentional scope and is reduced when attention is focused on task-related cues, for example during performance monitoring. This interpretation is also consistent with the present finding that supramarginal gyrus activation is at its highest when both performance monitoring and goal maintenance demands are low, and deactivates when either or both processes are engaged.

To examine goal maintenance, the task featured two different “go” responses that alternated between blocks as either pushing or pulling a lever. To succeed, participants were required to maintain in memory the relevant instruction. Goal maintenance was manipulated by the presence or absence of that instruction (“push” or “pull”) on the screen throughout the block. We found that goal maintenance recruited activity in the superior precentral gyrus (BA6). These data add converging evidence to the view that the superior aspect of the left precentral gyrus is involved in planning for goal-oriented behavior [51]–[53]. The fact that activity is enhanced under increased goal maintenance suggests that this region is sensitive to contextual demand and adapts accordingly in service of ongoing goals. Furthermore, the increased activation during sustained goal maintenance is consistent with the notion that this region is involved in maintaining a response set across trials and not simply initiating a motor response when necessary.

In contrast, a subset of the presupplementary motor area voxels that was active during response inhibition (i.e., no-go > go) was *less* active when goal maintenance demands were high compared to when they were low. Though the preSMA (and dACC) was generally active during response inhibition, high goal maintenance demand seems to have produced interference in this region. It is possible that the visual display of the instruction on the screen to “push” or “pull” (during low-maintenance blocks) generated increased response conflict on no-go trials compared to when the instruction was represented internally only (during high-maintenance blocks). If this were the case, it would suggest that one way to increase preSMA activity (and presumably the concordant conflict signal) would be to reduce goal maintenance demands by enhancing visual reminders of the task instructions instead of relying upon those instructions to be recalled and represented in working memory. For example, smokers attempting to quit might be aided in their real-life attempts at response inhibition if their goal to quit was made visually salient (e.g., on an electronic device) at times when cravings were known to be high such as in the morning.

### Limitations

One may wonder how well the simple neurocognitive task employed in the present study models the intended components of complex, real-world goal pursuit. For example, does the simple presence or absence of an instruction adequately manipulate goal maintenance? Though there are several ways of cognitively representing the goal on the task (e.g., “to succeed,” “to not push/pull the lever when I see an X”, “to push/pull for most letters”) the instruction to push or pull is the only one that changes across blocks making it both salient and relevant to success on the task. In other words, participants always had the abstract goal of “success”, and this goal was only served on some blocks with the subordinate goal of “pushing”, making maintaining that goal particularly important. To the extent that participants desired to be successful on the task (and the behavioral responses suggest that they did), keeping in mind whether to push or pull was critical to that goal. Whether the target instructions were displayed on the screen thus seems to be a relevant and direct manipulation of goal maintenance demands.

Similarly, it is important to consider the extent to which the comparison between go trials in go-only blocks and go trials in no-go blocks captures performance monitoring. Any differences in neural activity between those two types of go trials are likely due to vigilance for the occurrence of no-go trials and not due to error monitoring per se, which is more likely to occur during no-go trials themselves because of the increased risk of error on those trials. Indeed, several recent studies have used similar comparisons to examine the “preparation cost” of monitoring for possible stopping, or “proactive inhibitory control” [5], [54]–[55], and found similar results to those presented here. To the extent that performance monitoring involves detecting discrepancies between a current state (e.g., the current response) and a desired state (e.g., the correct response), and given that the need for vigilance for discrepancy is greater when the probability of committing an error is increased, we believe that the comparison between go trials in go-only and no-go blocks captures performance monitoring in the present task. This manipulation is especially attractive for the present purposes because performance monitoring can be varied independently from goal maintenance, allowing for a test of interactive neural systems. Future studies can build on these results by expanding performance monitoring even further to include longer time frames and explicit comparisons between the current and end states.

Finally, we recognize that the design is necessarily imbalanced because performance monitoring was only possible in the high response inhibition blocks (i.e., those with both go and no-go trials). The use of the go/no-go paradigm further restricts the number of response inhibition trials because they must be far less frequent than the “go” trials. Also, the uneven numbers of “go” trials between low- and high-monitoring blocks, and the difference in jittering between these blocks, is a limitation of this task design. However, these limitations were considered carefully in our design and we were able to achieve sufficient power to detect interaction effects. Other studies are currently underway in our lab to study goal process interaction effects using different tasks (each of which have their own limitations), with the hope that we may be able to produce convergent evidence for the results demonstrated here.

### Conclusion

In summary, the present study employed a novel task to investigate the simultaneous effects of three neurocognitive components of goal pursuit. Goal maintenance, performance monitoring, and response inhibition recruited a broad network of prefrontal, parietal, and subcortical structures. The components interacted to alter the neural response in a subset of those structures. These interactions suggest a failure of the “pure insertion” assumption when combining goal maintenance demands with inhibitory control or performance monitoring demands. This failure has implications for both construct and ecological validity in cognitive neuroscience. On the one hand, knowing that cognitive processes can interact with one another to influence neural activity suggests that extra care must be taken to isolate processes from one another to ensure that brain-mapping efforts are a faithful representation of the processes of interest. On the other hand, knowing that these processes often overlap in the real world suggests the need for more realistic task designs to ensure ecological validity of our neural results. We believe that this study provides a model for and first step toward unpacking the interacting mechanisms of higher-order goal pursuit in humans.

## References

[1] Oettingen G, Pak H, Schnetter K (2001). Self-regulation of goal setting: turning free fantasies about the future into binding goals.. Journal of Personality and Social Psychology.

[2] Pham LB, Taylor SE (1999). From Thought to Action: Effects of Process-Versus Outcome-Based Mental Simulations on Performance.. Personality and Social Psychology Bulletin.

[3] Berkman ET, Lieberman MD (2009). The neuroscience of goal pursuit: Bridging gaps between theory and data. The Psychology of Goals.. New York, NY: Guilford Press.

[4] Aharonovich E, Nunes E, Hasin D (2003). Cognitive impairment, retention and abstinence among cocaine abusers in cognitive-behavioral treatment.. Drug and Alcohol Dependence.

[5] Chikazoe J, Jimura K, Hirose S, Yamashita K-I, Miyashita Y (2009). Preparation to Inhibit a Response Complements Response Inhibition during Performance of a Stop-Signal Task.. Journal of Neuroscience.

[6] Dikman ZV, Allen JJ (2000). Error monitoring during reward and avoidance learning in high- and low-socialized individuals.. Psychophysiology.

[7] Muraven M (2010). Building self-control strength: Practicing self-control leads to improved self-control performance.. Journal of Experimental Social Psychology.

[8] Oettingen G, Honig G, Gollwitzer PM (2000). Effective self-regulation of goal attainment International Journal of Educational Research.

[9] Zimmerman BJ, Bandura A, Martinez-Pons M (1992). Self-Motivation for Academic Attainment: The Role of Self-Efficacy Beliefs and Personal Goal Setting.. American Educational Research Journal.

[10] Cabeza R, Nyberg L (2000). Imaging cognition II: An empirical review of 275 PET and fMRI studies.. Journal of Cognitive Neuroscience.

[11] Miller EK, Cohen JD (2001). An integrative theory of prefrontal cortex function.. Annual Review of Neuroscience.

[12] Stroop JR (1935). The basis of Ligon’s theory.. American Journal of Psychology.

[13] MacLeod C, Mathews A (1991). Biased cognitive operations in anxiety: Accessibility of information or assignment of processing priorities?. Behavioral Research and Therapy.

[14] MacDonald AW, Cohen JD, Stenger VA, Carter CS (2000). Dissociating the role of the dorsolateral prefrontal and anterior cingulate cortex in cognitive control.. Science.

[15] Baker SC, Frith CD, Frackowiak RSJ, Dolan RJ (1996). Active Representation of Shape and Spatial Location in Man.. Cerebral Cortex.

[16] Fletcher PC, Shallice T, Dolan RJ (1998). The functional roles of prefrontal cortex in episodic memory I. Encoding.. Brain.

[17] Banich MT, Milham MP, Atchley R, Cohen NJ, Webb A (2000). fMRI Studies of Stroop Tasks Reveal Unique Roles of Anterior and Posterior Brain Systems in Attentional Selection.. Journal of cognitive neuroscience.

[18] Herd SA, Banich MT, OReilly RC (2006). Neural Mechanisms of Cognitive Control: An Integrative Model of Stroop Task Performance and fMRI Data.. Journal of Cognitive Neuroscience.

[19] Bunge SA, Kahn I, Wallis JD, Miller EK, Wagner AD (2003). Neural Circuits Subserving the Retrieval and Maintenance of Abstract Rules.. Journal of Neurophysiology.

[20] Owen AM, McMillan KM, Laird AR, Bullmore E (2005). N-back working memory paradigm: A meta-analysis of normative functional neuroimaging studies.. Human Brain Mapping.

[21] Botvinick MM, Braver TS, Barch DM, Carter CS, Cohen JD (2001). Conflict monitoring and cognitive control.. Psychological Review.

[22] Botvinick MM, Cohen JD, Carter CS (2004). Conflict monitoring and anterior cingulate cortex: an update.. Trends in Cognitive Sciences.

[23] Van Veen V, Carter CS (2002). The timing of action-monitoring processes in the anterior cingulate cortex.. Journal of Cognitive Neuroscience.

[24] Carter CS, Macdonald AM, Botvinick M, Ross LL, Stenger VA (2000). Parsing executive processes: strategic vs. evaluative functions of the anterior cingulate cortex.. Proceedings of the National Academy of Sciences USA.

[25] Kerns JG, Cohen JD, MacDonald AW, Cho RY, Stenger VA (2004). Anterior cingulate conflict monitoring and adjustments in control.. Science.

[26] Taylor SF, Stern ER, Gehring WJ (2007). Neural systems for error monitoring: Recent findings and theoretical perspectives.. Neuroscientist.

[27] Aron AR (2007). The neural basis of inhibition in cognitive control.. Neuroscientist.

[28] Aron AR (2008). Progress in Executive-Function Research.. Current Directions in Psychological Science.

[29] Xue G, Aron AR, Poldrack RA (2008). Common neural substrates for inhibition of spoken and manual responses.. Cerebral Cortex.

[30] Aron AR, Robbins TW, Poldrack RA (2004). Inhibition and the right inferior frontal cortex.. Trends in Cognitive Sciences.

[31] Aron AR, Fletcher PC, Bullmore ET, Sahakian BJ, Robbins TW (2003). Stop-signal inhibition disrupted by damage to right inferior frontal gyrus in humans.. Nature Neuroscience.

[32] Aron AR, Monsell S, Sahakian BJ, Robbins TW (2004). A componential analysis of task-switching deficits associated with lesions of left and right frontal cortex.. Brain.

[33] Brown SM, Manuck SB, Flory JD, Hariri AR (2006). Neural Basis of Individual Differences in Impulsivity: Contributions of Corticolimbic Circuits for Behavioral Arousal and Control.. Emotion.

[34] Horn NR, Dolan M, Elliott R, Deakin JFW, Woodruff PWR (2003). Response inhibition and impulsivity: an fMRI study.. Neuropsychologia.

[35] Aron AR, Behrens TE, Smith S, Frank MJ, Poldrack RA (2007). Triangulating a cognitive control network using diffusion-weighted magnetic resonance imaging (MRI) and functional MRI.. Journal of Neuroscience.

[36] Leung H-C, Cai W (2007). Common and differential ventrolateral prefrontal activity during inhibition of hand and eye movements.. Journal of Neuroscience.

[37] Simmonds DJ, Pekar JJ, Mostofsky SH (2008). Meta-analysis of Go/No-go tasks demonstrating that fMRI activation associated with response inhibition is task-dependent.. Neuropsychologia.

[38] Wager TD, Sylvester C-YC, Lacey SC, Nee DE, Franklin M (2005). Common and unique components of response inhibition revealed by fMRI.. Neuroimage.

[39] Fuster JM (2008). The Prefrontal Cortex.. Boston, MA: Academic Press/Elsevier.

[40] Hampshire A, Chamberlain SR, Monti MM, Duncan J, Owen AM (2010). The role of the right inferior frontal gyrus: inhibition and attentional control.. NeuroImage.

[41] Dodds CM, Morein-Zamir S, Robbins TW (2011). Dissociating inhibition, attention, and response control in the frontoparietal network using functional magnetic resonance imaging.. Cerebral Cortex.

[42] Sharp DJ, Bonnell V, De Boissezon X, Beckmann CF, James SG (2010). Distinct frontal systems for response inhibition, attentional capture, and error processing.. Proceedings of the National Academy of Sciences.

[43] Verbruggen F, Aron AR, Stevens MA, Chambers CD (2010). Theta burst stimulation dissociates attention and action updating in human inferior frontal cortex.. Proceedings of the National Academy of Sciences.

[44] Venkatraman V, Rosati AG, Taren AA, Huettel SA (2009). Resolving response, decision, and strategic control: evidence for a functional topography in dorsomedial prefrontal cortex.. Journal of Neuroscience.

[45] Friston KJ, Price CJ, Fletcher P, Moore C, Frackowiak RS (1996). The trouble with cognitive subtraction.. Neuroimage.

[46] Menon V, Adleman N, White C, Glover G, Reiss A (2001). Error-related brain activation during a Go/NoGo response inhibition task.. Human Brain Mapping.

[47] Berkman ET, Falk EB, Lieberman MD (2011). In the trenches of real-world self-control: Neural correlates of breaking the link between craving and smoking.. Psychological Science.

[48] Gusnard DA, Raichle ME (2001). Searching for a baseline: functional imaging and the resting human brain.. Nature Reviews Neuroscience.

[49] Spreng RN, Mar RA, Kim ASN (2009). The common neural basis of autobiographical memory, prospection, navigation, theory of mind, and the default mode: a quantitative meta-analysis.. Journal of Cognitive Neuroscience.

[50] Shulman GL, Astafiev SV, McAvoy MP, dAvossa G, Corbetta M (2007). Right TPJ Deactivation during Visual Search: Functional Significance and Support for a Filter Hypothesis.. Cerebral Cortex.

[51] Grafton ST, Hamilton AFdC (2007). Evidence for a distributed hierarchy of action representation in the brain.. Human Movement Science.

[52] Johnson-Frey SH (2004). The neural bases of complex tool use in humans.. Trends in Cognitive Sciences.

[53] Toni I, Thoenissen D, Zilles K (2001). Movement Preparation and Motor Intention.. Neuroimage.

[54] Aron AR (2011). From reactive to proactive and selective control: Developing a richer model for stopping inappropriate responses.. Biological Psychiatry.

[55] Jahfari S, Stinear CM, Claffey M, Verbruggen F, Aron AR (2010). Responding with restraint: What are the neurocognitive mechanisms?. Journal of Cognitive Neuroscience.

